# Cell‐Type‐Specific WTAP and ALKBH5‐Mediated m^6^A Methylation Orchestrates Mental Disorders via Gut‐Brain Axis Metabolite Signaling: Multi‐Omics Evidence and Pyroptosis‐Associated Loop Mechanism

**DOI:** 10.1002/cns.70840

**Published:** 2026-03-15

**Authors:** Jingjing Zhang, Xue Liu, Xiaoming Liu, Liqun Zhao, Junjie Bi

**Affiliations:** ^1^ Qilu Aerospace Information Research Istitute Jinan China; ^2^ Department of Respiratory Medicine Affiliated Hospital of Shandong University of Traditional Chinese Medicine Jinan China; ^3^ Geriatric Medicine Center Affiliated Hospital of Shandong University of Traditional Chinese Medicine Jinan China

**Keywords:** CSF metabolites, gut microbiota, m^6^A modification, mendelian randomization, pyroptosis, single‐cell eQTL

## Abstract

**Introduction:**

Mental disorders affect one billion people and contribute 5% of global DALYs. Although N6‐methyladenosine (m^6^A) modification, gut microbiota, cerebrospinal fluid (CSF) metabolites, and pyroptosis have been implicated in disease pathogenesis, the directionality and magnitude of causal links remain elusive. We integrated two‐sample Mendelian randomization (MR) with single‐cell eQTL data to generate hypotheses regarding these relationships.

**Methods:**

Using large‐scale GWAS summary statistics, we estimated the causal effects of m^6^A regulators, gut microbiota, CSF metabolites, and pyroptosis on depression, schizophrenia (SZ), bipolar disorder (BD), attention‐deficit/hyperactivity disorder (ADHD), and post‐traumatic stress disorder (PTSD). Mediation analysis quantified hypothesized microbiota‐ and metabolite‐mediated proportions; single‐cell eQTL from 14 immune subsets localized genetic associations to cell types.

**Results:**

MR analysis suggested distinct causal associations of m^6^A modifiers (WTAP/ALKBH5) with mental disorders. Gut microbiota (e.g., GCA‐900066755, Bacillus) and CSF metabolites (e.g., theophylline, isoleucine) were estimated to potentially mediate 5.06%–45.28% of these effects, forming pathways such as WTAP → GCA‐900066755 → theophylline → ADHD and ALKBH5 → CAG‐390 → isoleucine → PTSD. These represent statistically inferred associations rather than experimentally validated pathways. Notably, these proportions were not based on multiple‐testing–corrected associations. Single‐cell eQTL mapping associated WTAP's ADHD risk with XCL1‐NK cells (OR = 1.445). Bidirectional MR suggested potential reciprocal associations between mental disorders and microbial/metabolite profiles. Pyroptosis demonstrated a tentative statistical association (3.99% explanatory power), though its specific biological role remains undefined.

**Conclusion:**

This multi‐omics integration generates a hypothesis regarding a potential m^6^A–microbiota–metabolite axis that may be associated with mental disorders, with pyroptosis as a potentially related peripheral factor. Should these associations prove causal, the identified relationships could suggest peripheral intervention nodes, while combinatorial targeting might provide directions for preventive psychiatry. Experimental validation is required to confirm the biological mechanisms implied by these genetic associations.

## Graphical abstract

MR analysis revealed distinct causal associations of m^6^A modifiers (WTAP/ALKBH5) with mental disorders. Gut microbiota (GCA‐900066755, Bacillus) and CSF metabolites (theophylline, isoleucine) mediated 5.06%–45.28% of effects via pathways such as WTAP → GCA‐900066755 → theophylline → ADHD. Single‐cell eQTL linked WTAP‐mediated ADHD risk to XCL1‐NK cells, while pyroptosis showed a tentative association (3.99% explanatory power).

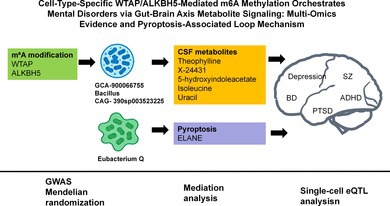

## 1 Introduction

Mental disorders constitute a heterogeneous group of conditions that profoundly perturb cognition, emotion and behavior. Major depressive disorder manifests as recurrent episodes of depressed mood, anhedonia and suicidal ideation, with lifetime recurrence approaching 90% [1, 2, 3]. Schizophrenia (SZ) is characterized by persistent hallucinations and persecutory delusions, coupled with affective flattening and cognitive impairment; suicide risk is 10–20 times that of the general population [4, 5, 6, 7, 8]. Bipolar disorder (BD), with heritability up to 70%, confers marked psychosocial impairment, lifetime recurrence near 90% and a twenty‐fold increase in suicide risk [9, 10]. Attention‐deficit/hyperactivity disorder (ADHD), a childhood‐onset neurodevelopmental syndrome, persists into adulthood in approximately 60% of cases, severely compromising academic and occupational functioning [11, 12, 13, 14]. Post‐traumatic stress disorder (PTSD) produces profound psychological distress and elevates the risk of cardiovascular disease and metabolic syndrome [15, 16]. The World Mental Health Report and Mental Health Atlas 2024 (WHO) estimate that over 1 billion people live with mental health disorders, which account for 5% of global disability‐adjusted life‐years (DALYs) and rank as the second leading cause of years lived with disability (YLDs), contributing one in six YLDs worldwide [17].

N6‐methyladenosine (m^6^A) is a dynamic, reversible epitranscriptomic mark that governs alternative splicing, nuclear export, translation, and degradation of mRNA across eukaryotes [18, 19, 20]. Emerging evidence links m^6^A dysregulation to delayed cerebral development, impaired axonal regeneration, and disrupted learning and memory, implicating the modification and its enzymatic machinery in the onset and progression of multiple psychiatric disorders [21, 22, 23].

Gut‐brain crosstalk is well recognized in health and disease, with the gut microbiota serving as a key communicator between these distant organs; nevertheless, the mechanisms by which microbes shape gut‐brain axis development and function remain largely unresolved [24]. Although accumulating evidence links gut microbiota to host health and to the pathogenesis of complex disorders, including psychiatric illness, the exceptional heterogeneity of microbial communities has precluded rigorous establishment of causal direction in the microbiota‐brain relationship [25, 26].

Cerebrospinal fluid (CSF) metabolites, by virtue of their anatomical and functional continuity with the central nervous system and their enrichment in low‐molecular‐weight metabolites that reflect real‐time neural biochemistry, provide an unrivaled window into CNS‐specific pathophysiology. Consequently, CSF metabolites profiling has become a cornerstone in the search for objective biomarkers and aetiological insights across mental disorders [27, 28, 29].

Pyroptosis is a caspase and gasdermin mediated, pro‐inflammatory form of programmed cell death that operates as an essential component of innate immunity [30]. Its functional duality is increasingly recognized: while moderate pyroptotic signaling recruits immune cells and amplifies host defense, excessive activation precipitates uncontrolled inflammation and collateral tissue injury [31]. Emerging evidence further implicates pyroptotic pathways in the pathogenesis of several mental disorders [32, 33].

Immune‐cell‐based expression quantitative trait locus (eQTL) analyses are increasingly used to dissect the pathogenesis of mental disorders [34]. Bulk‐tissue approaches, however, blur spatial, temporal, and cell‐type resolution, limiting the precision of earlier findings [35, 36]. Recent advances in single‐cell genomics now enable gene‐expression–disease associations to be mapped at cell‐type resolution, facilitating the discovery of novel therapeutic targets [37, 38].

To systematically dissect the causal relationships among m^6^A modification, gut microbiota, CSF metabolites, and pyroptosis in mental disorders, we conducted a comprehensive two‐sample Mendelian randomization (MR) analysis. This method leverages genetic variants as instrumental variables to infer causality between exposures and complex traits while minimizing confounding and reverse causation inherent in observational studies [39, 40]. We first quantified the causal impact of the gut microbiome, m^6^A modification, CSF metabolites, and pyroptosis on five mental disorders (depression, SZ, BD, ADHD, and PTSD), and then tested whether gut microbiota mediate the m^6^A–mental disorder pathway and whether CSF metabolites and pyroptosis act as downstream mediators of the microbiota–mental disorder axis. Bidirectional MR was further applied to assess whether genetic liability to mental disorders reciprocally shapes the microbial community and CSF metabolite profile. To locate the genes underpinning these molecular layers, we leveraged single‐cell immune eQTL data covering 14 distinct cell types to dissect how each gene modulates disease risk within individual immune compartments.

## 2 Methods

### 2.1 Study Design

**FIGURE 1:**
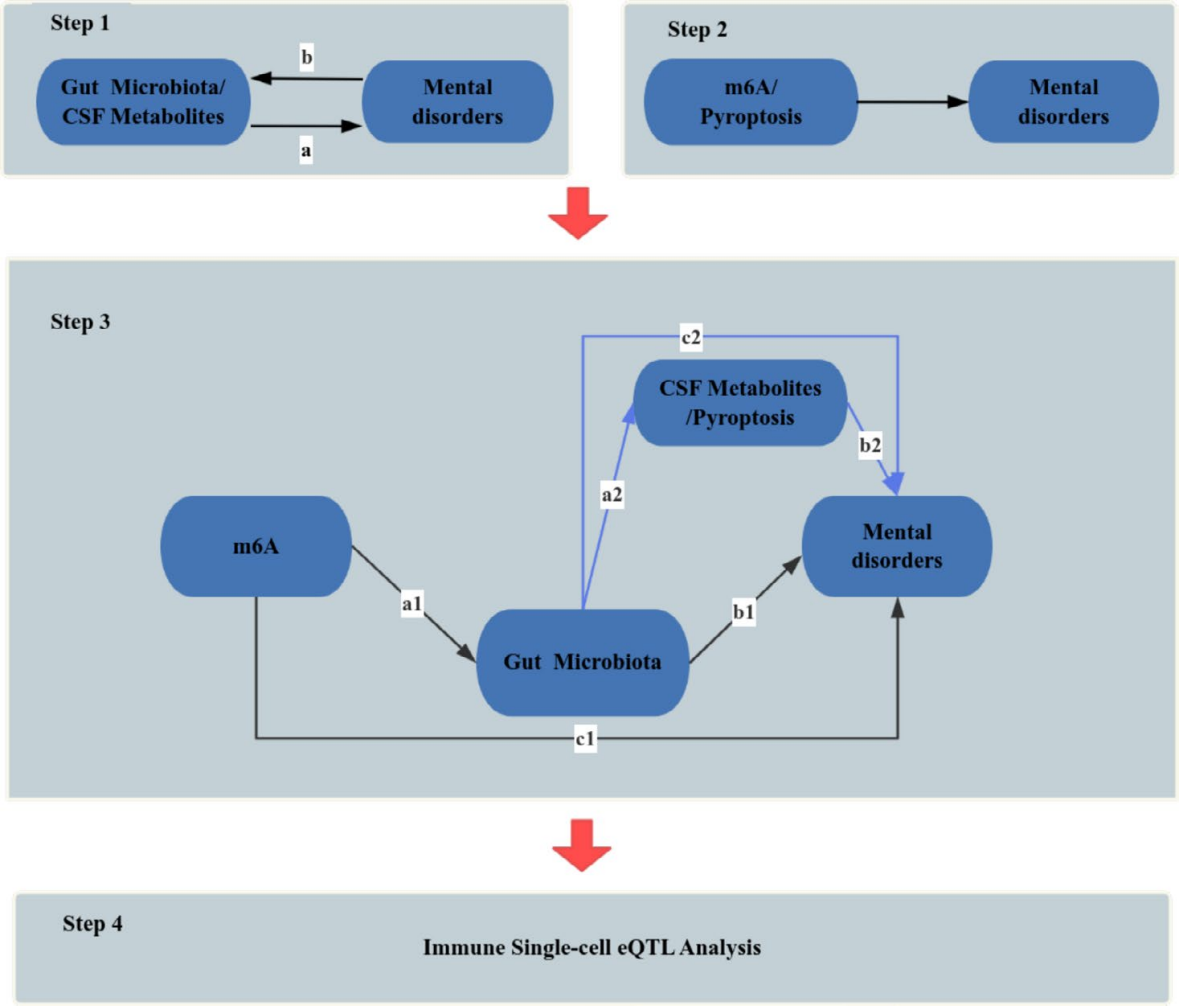
Basic idea of the research. Step 1a maps the unidirectional influence of gut microbiota and CSF metabolites on mental disorders, while Step 1b reverses this directionality to assess reciprocal causation. Step 2 investigates how m^6^A and pyroptosis independently contribute to mental disorders pathogenesis. Step 3 deconstructs the mediating pathways: The m^6^A–gut microbiota–mental disorders (c1 total effect, a1 microbiota shift, b1 mental disorders outcome) and the microbiota–CSF metabolites/pyroptosis–mental disorders axis (c2 total effect, a2 CSF metabolites/pyroptosis induction, b2 mental disorders consequence). Step 4 leveraged immune single‐cell eQTL data from 14 cell types to dissect how the differentially expressed genes identified in Step 2 influence disease within each immune compartment.

Figure 1 outlines the four‐stage design. First, the causal contribution of gut microbiota and CSF metabolites to mental disorders was interrogated (Step 1a). Next, the possible regulatory roles of m^6^A modification and pyroptosis in mental disorders were investigated (Step 2). Step 3 then dissected the putative mediation chains: m^6^A‐microbiota‐mental disorders and microbiota‐CSF metabolites/pyroptosis‐mental disorders. To test for reverse causation, bidirectional MR analyses between microbiota and mental disorders were additionally performed (Step 1b). Step 4 leveraged immune single‐cell eQTL data from 14 cell types to dissect how the differentially expressed genes identified in Step 3 influence disease within each immune compartment.

All MR estimates exploited single‐nucleotide polymorphisms (SNPs) as instrumental variables under the standard trio of assumptions: (i) robust SNP–exposure association, (ii) independence from confounders, and (iii) exclusion of direct SNP–outcome pathways [41].

### 2.2 Data Source

#### 2.2.1 Source of Gut Microbiota Data

Genetic instruments for the gut microbiome were extracted from the 2022 meta‐GWAS, which integrated questionnaire data, self‐reports and shotgun metagenomics of 5959 individuals (Nat Genet 2022). After quality control, 7.97 M SNPs were tested against 2801 taxonomic features, yielding 473 GTDB‐level taxa with genome‐wide significance [42]. Summary‐level results are publicly available through the GWAS Catalog (accession block GCST90032172–GCST90032235).

#### 2.2.2 Source of CSF Metabolites Data

CSF metabolome data were drawn from a multicenter investigation that pooled two longitudinal AD cohorts with both CSF metabolomics and genotyping: the Wisconsin Alzheimer's Disease Research Center and the Wisconsin Registry for Alzheimer's Prevention. To maximize generalizability, only cognitively healthy participants were retained. In total, 689 individuals (532 from WADRC and 168 from WRAP) contributed CSF samples, yielding a final panel of 338 rigorously quantified metabolites [43]. Summary‐level results are publicly available through the GWAS Catalog (accession block GCST90026002‐GCST90026289).

#### 2.2.3 Source of m^6^A and Pyroptosis Data

Data on m^6^A and pyroptosis‐related genes were extracted from multiple studies. In total, 19 m^6^A genes [44, 45, 46, 47] and 33 pyroptosis genes [48, 49] were compiled (Table S1).

#### 2.2.4 Source of eQTL and pQTL Data

Cis‐eQTL data for all genes examined in this study were downloaded from the eQTLGen consortium portal (https://www.eqtlgen.org/cis‐eqtls.html) [50]; all datasets correspond to cis‐acting expression quantitative trait loci derived exclusively from whole‐blood samples.

Original pQTL data were obtained from the deCODE database (https://www.decode.com/summarydata/) [51], comprising 4907 individual files derived exclusively from the Icelandic population. We then extracted cis‐pQTLs according to the following criteria: (1) the SNP and the gene reside on the same chromosome; (2) the SNP is located within ±1 Mb of the gene. In total, 4706 cis‐pQTL files were obtained.

#### 2.2.5 Source of Immune Single‐Cell eQTL Data

Immune single‐cell eQTL data were obtained from the OneK1K study, which generated single‐cell RNA‐seq profiles of 1.27 million PBMCs from 982 donors. The consortium established a cell‐classification framework and, by integrating these transcriptomes with genotype data, mapped cis‐genetic effects on gene expression across 14 immune cell types, identifying 26,597 independent cis‐eQTLs [52].

#### 2.2.6 Source of Mental Disorders Data

Genetic associations for the mental disorders were extracted from the FinnGen R11 release [53].

The present work is a secondary analysis of publicly available genome‐wide summary statistics. All original genome‐wide association studies were approved by their respective institutional review boards. Because only de‐identified, aggregated data were used, no additional ethical clearance or individual consent was required. Detailed information is provided in Table 1.

**TABLE 1:** Detailed information for the data source.

Trait	Gwas ID	Population	Year	Sample size
Gut microbiota	GCST90032172‐GCST90032235	European	2022	5959
CSF metabolites	GCST90026002‐GCST90026289	American	2021	689
Immune single‐cell eQTL	/	European	2022	982
Depression	F5_DEPRESSION_RECURRENT	European	2023	22,768/236510
Schizophrenia (SZ)	F5_SCHZPHR	European	2023	6933/439144
Bipolar affective disorders (BD)	F5_BIPO	European	2023	8209/394756
Disturbance of activity and attention (ADHD)	F5_ADHD	European	2023	3702/445327
Post‐traumatic stress disorder (PTSD)	F5_PTSD	European	2023	3005/403817

### 2.3 Instrumental Variables Selection

#### 2.3.1 m^6^A Genes

We selected cis‐eQTL instruments (SNPs) using the following criteria: *p* < 5 × 10^−8^, clumping window = 10,000 kb, *r* ^2^ < 0.1. After filtering, 15,695 genes retained valid cis‐eQTL data. Intersecting this gene set with the 19 m^6^A‐related genes yielded 12 m^6^A genes with usable cis‐eQTL information.

#### 2.3.2 Pyroptosis Genes

We screened the 4706 cis‐pQTL files for instrumental SNPs using *p* < 5 × 10^−8^, a clumping window of 10,000 kb, and *r* ^2^ < 0.1, yielding 1615 cis‐pQTL datasets. Intersection of these datasets with 33 pyroptosis‐related genes identified 11 pyroptosis genes with valid cis‐pQTL data.

#### 2.3.3 Gut Microbiota and CSF Metabolites

For gut microbiota and CSF metabolites we first clumped SNPs at *p* < 5 × 10^−6^, *r* ^2^ < 0.001 and a 10,000 kb window. After harmonizing the effect alleles between exposure and outcome datasets, palindromic A/T or G/C variants were removed. The proportion of phenotypic variance explained (*R* ^2^) and the per‐SNP F‐statistic were then calculated; any locus with *F* < 10 was discarded to avoid weak‐instrument bias. In light of the significant sample size imbalance between the CSF metabolome data (*n* = 689) and disease outcomes (*n* > 200,000), we adopted rigorous instrument selection criteria, preserving only genetic variants with average *F*‐statistics greater than 20. This robust instrument property sufficiently addresses potential weak instrument bias arising from the modest exposure sample size, thus guaranteeing reliable causal inference. Full instrument descriptors are supplied in Tables S2–S5.

### 2.4 MR Analysis

Two‐sample MR was used to estimate the causal effects of (i) gut microbiota, (ii) CSF metabolites, (iii)m^6^A and (iv) pyroptosis on mental disorders.

(steps 1a and 2 in Figure 1). Inverse‐variance weighted (IVW) regression was the primary estimator; for traits represented by a single SNP, the Wald ratio is reported [54].

Effects are expressed as odds ratios (ORs) with 95% confidence intervals (CIs). An IVW *p*‐value < 0.05 was used as the preliminary screening criterion. After Bonferroni correction for multiple testing, the significance thresholds for gut microbiota, CSF metabolites, m^6^A genes, and pyroptosis genes for mental disorders were set at 1.057 × 10^−4^ (0.05/473), 1.479 × 10^−4^ (0.05/338), 4.167 × 10^−3^ (0.05/12), and 4.454 × 10^−3^ (0.05/11), respectively. Associations with *p* < 0.05 but failing to reach the corresponding Bonferroni‐corrected thresholds were regarded as suggestive of an association.

### 2.5 Bidirectional Causality Analysis

To examine whether mental disorders reciprocally shape the gut microbiota and CSF metabolites, we swapped the exposure and outcome variables so that mental disorders‐associated variants served as instruments (steps 1b, Figure 1). SNPs were retained at genome‐wide suggestive significance (*p* < 5 × 10^−6^) after clumping.

### 2.6 Sensitivity Analysis

We performed four categories of sensitivity analyses, consisting of five independent statistical tests, to ensure the robustness of the MR results. The specific analyses and tests are as follows: (i) Heterogeneity assessment: Cochran's Q test under both IVW and MR‐Egger frameworks (2 tests); (ii) Directional pleiotropy test: MR‐Egger intercept test (1 test); (iii) Outlier detection: MR‐PRESSO global test (1 test); (iv) Influence diagnostics: leave‐one‐out analysis (1 test) [55].

Analytical workflows were implemented in R 4.4.2. Core MR estimates were generated with the TwoSampleMR package. This study follows the STROBE‐MR statement, with the detailed checklist available Table S14.

### 2.7 Mediation Analysis

In the mediation analysis (step 3 in Figure 1) we examined m^6^A modification, gut microbiota, CSF metabolites and pyroptosis because of their established influence on psychiatric disorders. To test whether microbiota mediate the m^6^A–disorder pathway, we first assumed that m^6^A variants causally affect microbial abundance (step 3a1). To determine whether CSF metabolites and pyroptosis mediate the microbiota–disorder pathway, we then assumed that microbiota causally influence CSF metabolites and pyroptosis (step 3a2).

### 2.8 Single‐Cell eQTL Analysis

Single‐cell eQTL summary statistics were first decomposed into gene‐ and cell‐type‐specific files restricted to genes retained in the Section 2.7 mediation analysis. Within each file, variants with *p* < 0.05 were LD‐clumped (window = 100 kb, *r* ^2^ ≥ 0.3) to retain independent lead SNPs. The resulting trimmed QTL list was used as the exposure in downstream MR analyses, with disease as the outcome.

## 3 Results

### 3.1 Causal Associations of Gut Microbiota and CSF Metabolites With Mental Disorders

MR analysis indicated statistically significant causal effects of gut microbiota and CSF metabolites on all five mental disorders. For depression, 27 gut microbial taxa (16 increasing risk, 11 protective) and 21 CSF metabolites (9 increasing risk, 12 protective) were identified. SZ was associated with 18 gut microbial taxa (4 increasing risk, 14 protective) and 9 CSF metabolites (5 increasing risk, 4 protective). BD showed causal associations with 21 gut microbial taxa (10 increasing risk, 11 protective) and 16 CSF metabolites (6 increasing risk, 10 protective). ADHD was linked to 16 gut microbial taxa (3 increasing risk, 13 protective) and 20 CSF metabolites (10 increasing risk, 10 protective). PTSD demonstrated associations with 9 gut microbial taxa (1 increasing risk, 8 protective) and 29 CSF metabolites (9 increasing risk, 20 protective).

**FIGURE 2:**
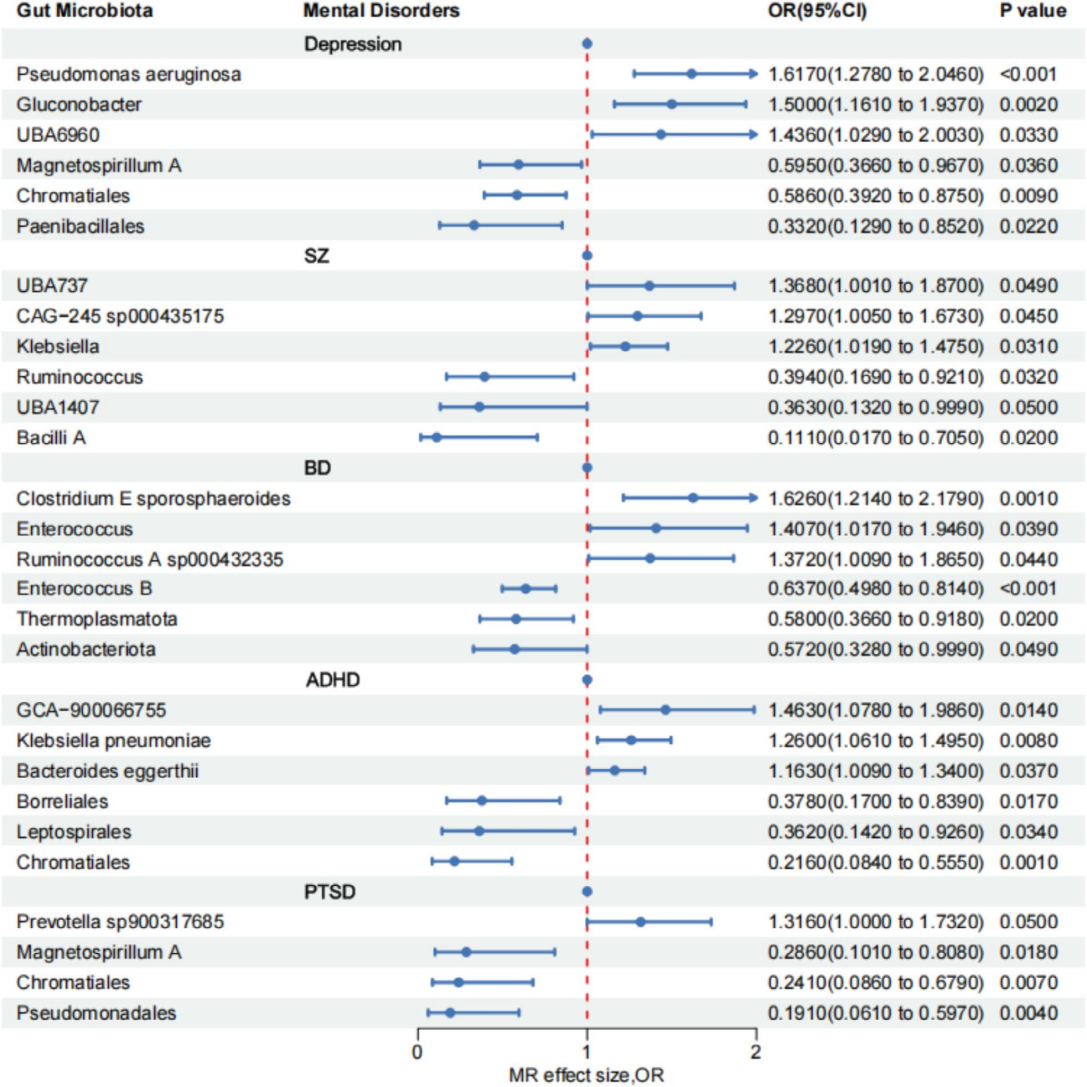
The forest plot between gut microbiota and mental disorders.

**FIGURE 3:**
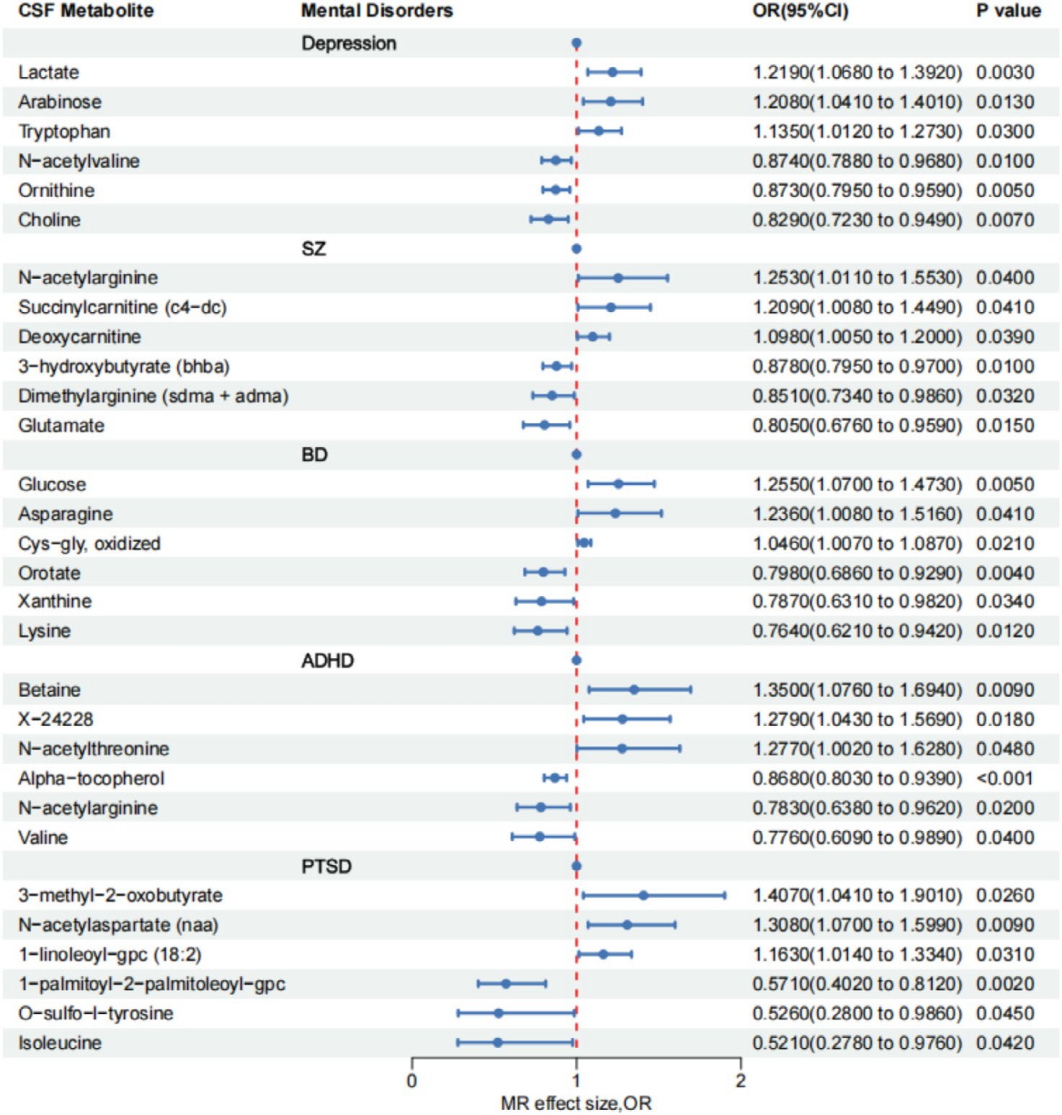
The forest plot between CSF metabolites and mental disorders.

Following Bonferroni correction, *Pseudomonas aeruginosa* remained significantly associated with depression (OR = 1.617, 95% CI = 1.278–2.046, *p* = 6.31 × 10^−5^) (Figures 2 and 3; Tables S6 and S7).

### 3.2 Causal Associations of m^6^A and Pyroptosis With Mental Disorders

**FIGURE 4:**
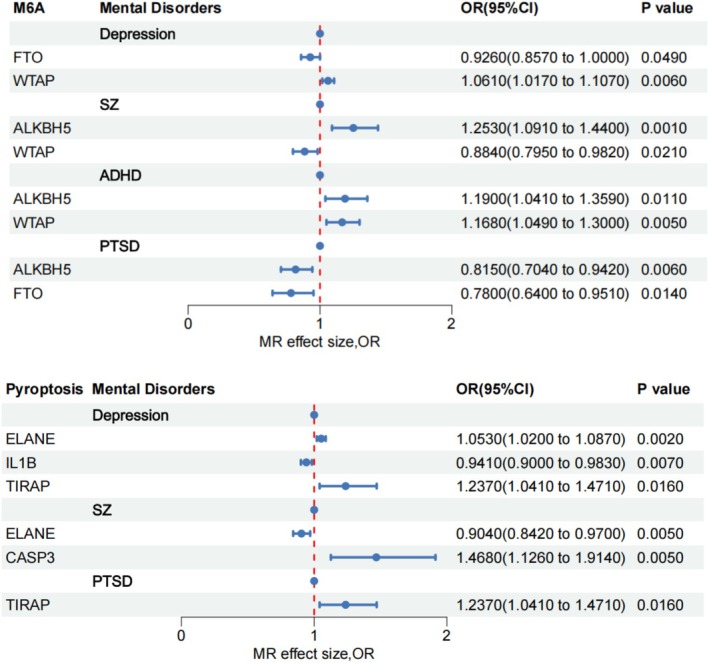
The forest plot between m^6^A, pyroptosis and mental disorders.

Three m^6^A genes exert distinct effects on each of four mental disorders, while four pyroptosis genes differentially influence each of three mental disorders (Figure 4; Tables S8 and S9).

After Bonferroni correction, m^6^A gene ALKBH5 remained associated with SZ (OR = 1.253, 95% CI = 1.091–1.440, *p* = 0.001), pyroptosis gene ELANE remained associated with Depression (OR = 1.053, 95% CI = 1.020–1.087, *p* = 0.002) (Figures 2 and 3; Tables S6 and S7).

### 3.3 Sensitivity Analysis

MR‐Egger intercept, MR‐PRESSO outlier, and Cochran's *Q* tests led to exclusion of three gut‐microbe estimates and 10 CSF‐metabolite estimates (Table S10). Leave‐one‐out analyses confirmed that the remaining signals were not driven by single influential SNPs.

### 3.4 Bi‐Directional Causal Effects of Mental Disorder on Gut Microbiota and CSF Metabolites

Treating psychiatric diagnosis as exposure revealed widespread effects on gut microbiota and CSF metabolites. At *p* < 0.05, depression predicted 22 microbial taxa and one CSF metabolite; schizophrenia modulated 18 microbial taxa and 16 CSF metabolites; bipolar disorder altered five microbial taxa and 18 CSF metabolites; ADHD reshaped 22 microbial taxa and 10 CSF metabolites; PTSD reshaped 11 microbial taxa and nine CSF metabolites (Tables S11 and S12). These bidirectional associations underscore the complex feedback loops that must be considered in mediation analyses.

### 3.5 Mediation Analysis

Gut microbiota mediates the pathway linking m^6^A modification to mental disorders. Specifically, GCA‐900066755 contributed 12.31% of the total causal proportion through the WTAP‐ADHD axis, while Bacillus accounted for 11.36% via the WTAP‐depression axis, and CAG‐390 sp003523225 contributed 9.70% through the ALKBH5‐PTSD axis.

CSF metabolites further transmitted microbiota signals: theophylline (9.04%) and X‐24431 (5.93%) mediated the GCA‐900066755‐ADHD axis; 5‐hydroxyindoleacetate (5.06%) modulated the Bacillus‐depression axis; and isoleucine (26.23%) and uracil (45.28%) dominated the CAG‐390 sp003523225‐PTSD axis.

Additionally, pyroptosis was identified as a mediator, where ELANE‐driven pyroptosis (4%) explained part of the Eubacterium Q‐depression axis effect, suggesting compensatory regulation of the primary pathway (Table 2).

**TABLE 2:** Mediation effect of m^6^A, gut microbiota, CSF metabolites, pyroptosis and mental disorders.

Exposure	Outcome	Mediator	Total effect	Direct effect A	Direct effect B	Mediation effect	Mediated proportion
WTAP	ADHD	GCA‐900066755	0.155	0.050	0.381	0.019	12.309%
WTAP	Depression	Bacillus	0.060	0.015	0.447	0.007	11.361%
ALKBH5	PTSD	CAG‐390 sp003523225	−0.205	0.056	−0.358	−0.020	9.704%
GCA‐900066755	ADHD	Theophylline	0.381	−0.509	−0.068	0.034	9.036%
GCA‐900066755	ADHD	X‐24431	0.381	−0.519	−0.043	0.023	5.929%
Bacillus	Depression	5‐hydroxyindoleacetate	0.447	−1.519	−0.015	0.023	5.064%
CAG‐390 sp003523225	PTSD	Isoleucine	−0.358	0.144	−0.652	−0.094	26.228%
CAG‐390 sp003523225	PTSD	Uracil	−0.358	−0.210	0.773	−0.162	45.276%
Eubacterium Q	Depression	ELANE	0.318	0.246	0.051	0.013	3.986%

*Note:* Total effect: The causal role of m^6^A (gut microbiota) on mental disorders. Direct effect A: The causal role of m^6^A on gut microbiota (gut microbiota on CSF metabolites/pyroptosis). Direct effect B: The causal role of gut microbiota (CSF metabolites/pyroptosis) on mental disorders. Mediation effect = Direct effect A **×** Direct effect B. Mediated proportion = Mediation effect/total effect.

Multi‐omic mediation analysis identified five significant four‐step chains in the order m^6^A → gut microbe → CSF metabolite → mental disorders: WTAP → GCA‐900066755 → theophylline → ADHD; WTAP → GCA‐900066755 → X‐24431 → ADHD; WTAP → Bacillus → 5‐hydroxyindoleacetate → depression; ALKBH5 → CAG‐390 → isoleucine → PTSD; and ALKBH5 → CAG‐390 → uracil → PTSD.

Individual metabolite‐specific mediation proportions ranged from 5% to 45%, indicating partial rather than exclusive or dominant mediation within a broader network of concurrent biological processes. Meanwhile, these proportions were not based on multiple‐testing–corrected associations.

### 3.6 Single‐Cell eQTL Analysis

WTAP selectively increased ADHD risk within XCL1‐NK cells (OR = 1.445, 95% CI = 1.129–1.850, *p* = 0.003) but showed no effect in the remaining 13 immune cell subsets. Neither ALKBH5 nor ELANE exerted measurable effects in any of the 14 immune cell types examined (Table S13).

## 4 Discussion

Systematic multi‐omics integration generates hypotheses suggesting that m^6^A modification, gut microbiota, CSF metabolites and pyroptosis may collectively associate with mental disorder susceptibility. Statistical analyses identified four‐node directional relationships consistent with hypothesized chains, such as m^6^A regulation (genetically proxied by WTAP, ALKBH5) → gut microbiota (GCA‐900066755, Bacillus) → CSF metabolites (theophylline, uracil, 5‐hydroxyindoleacetate) → mental disorders (ADHD, depression, PTSD), though these represent statistically inferred associations rather than experimentally validated biological pathways. These findings invite reconsideration of conventional paradigms that posit mental disorders arise solely from intracerebral molecular dysregulation, instead proposing a peripheral epigenetic‐microbiota‐metabolic axis as a potential modulatory system warranting functional validation. The identified associations constitute genetically predicted links between m^6^A modification and mental disorders, awaiting mechanistic confirmation. Estimated metabolite‐specific mediation proportions varied (5.06%–45.28%), suggesting differential pathway hypothesized contributions across disorders. While these statistical findings provide a theoretical framework for investigating peripheral interventions (e.g., gut microbiota modulation or metabolite supplementation), experimental validation is required before therapeutic application, potentially informing future subtype‐specific precision intervention strategies.

### 4.1 Causal Effects of WTAP‐ and ALKBH5‐Mediated m^6^A Modifications on Mental Disorders

Elevated WTAP expression is associated with aggravated ADHD‐like behaviors, whereas increased ALKBH5 expression correlates with attenuated PTSD‐like phenotypes, indicating that m^6^A modifications differentially regulate distinct psychiatric disorders. Although WTAP lacks intrinsic catalytic activity, it serves as a critical regulatory subunit of the m^6^A methyltransferase complex and enhances METTL3/METTL14‐mediated deposition of m^6^A on transcripts involved in neurotransmission or inflammation [56, 57, 58]. This modification can reduce transcript stability or impair translational efficiency, thereby disrupting neurotransmitter homeostasis and synaptic plasticity and exacerbating ADHD or depression‐like phenotypes [59]. Conversely, ALKBH5, an m^6^A demethylase, attenuates PTSD‐like phenotypes by removing m^6^A marks from glial pro‐inflammatory or glutamate‐receptor mRNAs, leading to transcript destabilization, suppressed neuro‐inflammation and excitotoxicity, and ultimately enhanced stress resilience that mitigates PTSD‐like behaviors [60, 61].

Recent murine studies show that germline or cell‐type‐specific deletion of WTAP or ALKBH5 alters synaptic‐protein translation and social behavior. Astrocytic ALKBH5 deletion reverses stress‐induced social avoidance and restores GLT‐1 expression, dendritic‐spine density, and calcium signaling [61]. Conversely, microglial WTAP promotes M1‐type polarization and neuroinflammation via the SIRT1/C/EBPβ axis [62]. Population‐level causal evidence has, however, remained unavailable.

Using eQTLs as instrumental variables, we provide the first causal estimate of m^6^A regulation and partition its total effect into a direct brain‐intrinsic component and an indirect gut‐microbiome–CSF‐metabolite‐mediated component. Single‐cell cis‐eQTL mapping further restricted the signal to XCL1‐NK cells, circumventing dilution bias inherent in bulk brain or blood derived datasets. XCL1‐NK cells secrete chemokines that can modulate neuroinflammation [63]; WTAP‐mediated m^6^A modification enhances their pro‐inflammatory activity, thereby increasing ADHD risk [64, 65]. In contrast, WTAP shows no detectable effect in other immune subsets, consistent with cell‐type‐specific neuroimmune regulation.

m^6^A modifications may shape gut microbiota composition through three distinct mechanistic layers: First, at the epithelial barrier level, WTAP/ALKBH5 may alter the physicochemical environment for microbial colonization by regulating the expression of tight junction proteins and antimicrobial peptides [66, 67, 68]. Secondly, at the immunoregulatory level, m^6^A modifications differentially modulate pro‐ and anti‐inflammatory factors within specific immune subsets (e.g., XCL1‐NK cells), generating either immune tolerance or clearance pressure against particular bacterial taxa, as evidenced by our single‐cell cis‐eQTL analysis [69, 70, 71, 72]. Finally, at the metabolic level, modulation of bile acid and mucin metabolic genes alters the availability of luminal nutrient substrates, thereby selecting for bacterial taxa with specific metabolic capabilities [73, 74, 75]. These three mechanistic layers do not operate in isolation; instead, they constitute a dynamic “host epigenetic‐microbiota co‐evolutionary” network.

### 4.2 Bidirectional Interactions and Mediatory Mechanisms of Gut Microbiota and CSF Metabolites in Mental Disorders

Among the microbial taxa identified in this study, some are consistent with previous reports (e.g., the mood‐modulating potential of Bacillus) [76, 77], whereas the majority are newly implicated in causal relationships with mental disorders. For instance, Chromatiales emerged as a protective factor against ADHD, depression and PTSD, an association not previously reported, and may do so by producing short‐chain fatty acids that tighten the blood–brain barrier [78]. *Staphylococcus aureus* Flemingii increased risk for BD while decreasing risk for SZ, underscoring that the same microbe can exert disease specific and directionally opposite effects on psychiatric illness.

CSF metabolites, acting as direct messengers in microbe–brain communication, exhibited robust causal effects. In ADHD, theophylline showed a protective association; theophylline, as a non‐selective adenosine‐receptor antagonist, may ameliorate symptoms by enhancing dopaminergic and noradrenergic tone [79, 80, 81]. 5‐hydroxyindoleacetate was protective in depression, yet prior evidence for 5‐hydroxyindoleacetate in mood disorders remains inconsistent. Mediation analyses showed metabolites act as partial conduits in the microbe‐to‐disease pathway (e.g., isoleucine 26.23% of total PTSD effect), implying parallel routes such as immune‐cell infiltration or pyroptosis.

Bidirectional MR showed that mental disorders reciprocally reshape microbial and metabolite profiles; for example, depression modulated 22 microbial taxa. Such feedback loops may account for the clinical co‐persistence of chronic illness and microbial dysbiosis, underscoring the need for interventions that simultaneously target psychosocial stressors and the biological microenvironment. Schizophrenia exhibited broad effects on CSF metabolites (16 associations), suggesting that either antipsychotic medication or the disease itself disrupts metabolic homeostasis; future studies must disentangle illness from treatment‐driven effects.

### 4.3 Pyroptosis‐Associated Regulatory Mechanisms

Although ELANE mediated pyroptosis explained only 4% of the Eubacterium Q–depression effect, this modest proportion probably reflects the inability of peripheral‐blood eQTLs to capture microglial pyroptosis within the brain. Viewed alongside robust genetic evidence implicating the NLRP3–GSDMD axis in depression and its central role in microglial pyroptosis [33, 82, 83, 84], our data indicate that central pyroptosis remains a hidden mediator that warrants further investigation.

### 4.4 Study Limitations

This study has several limitations.

First, the causal inferences drawn from this MR study are statistical rather than mechanistic. MR cannot elucidate the intermediate molecular, cellular, or physiological processes linking the genetic variant to the phenotype. Therefore, the proposed directional relationships (e.g., WTAP → gut microbiota → metabolite → ADHD) represent hypothesized causal chains inferred from genetics, not experimentally confirmed biological pathways.

Second, mediation analysis suggested that gut microbiota and CSF metabolites may mediate 5.06%–45.28% of the genetic effects. It should be noted that these proportions represent estimates of partial mediation, implying the existence of additional parallel mechanisms beyond the identified pathways (including other microbial taxa, distinct metabolite categories, and direct gene regulatory effects). Due to sample size limitations, the specific values of these proportion estimates should be interpreted with caution. Furthermore, given that these mediation analysis results were not corrected for multiple testing, there is a risk of false positives; thus, these findings require validation in subsequent independent studies.

Third, although *F*‐statistics > 20 indicate relatively strong instrument strength, weak instrument bias cannot be completely excluded given the large sample size disparity between the CSF metabolome and disease GWAS (*n* = 689 versus *n* > 200,000). As *F*‐statistics are calculated in the discovery cohort, they may be inflated by the “winner's curse,” potentially overestimating the true instrument strength and biasing effect estimates toward the null hypothesis (attenuation bias).

Fourth, eQTL data were derived predominantly from individuals of European ancestry; extrapolation to Asian, African, or other populations should be undertaken cautiously.

### 4.5 Future Research Directions

Building on our findings, future work should prioritize 3 main areas.

Stage i: In vitro validation of the causal regulatory relationship between WTAP, ALKBH5, and GCA‐900066755, Bacillus, CAG‐390 sp003523225, employing co‐culture systems or transcriptomic analysis to elucidate molecular interactions.

Stage ii: Functional validation of pathway mechanisms underlying WTAP, ALKBH5‐mediated microbiota regulation in gene‐edited mouse models (WTAP, ALKBH5 knockdown or knockout), characterizing alterations in gut microbiota composition and metabolite profiles following experimental interventions.

Stage iii: Clinical validation through collection of primary fecal samples alongside optional cerebrospinal fluid from patients, investigating statistical correlations between WTAP, ALKBH5, and gut microbiota‐metabolite dynamics.

## 5 Conclusion

Large‐scale genomic and multi‐omics integration provides statistical evidence supporting a hypothesized m^6^A → gut microbiota → CSF metabolite → mental disorder associative axis, which identifies candidate four‐node directed pathways for experimental validation. Analyses suggest central pyroptosis may represent an additional potential mediator whose contribution may be underestimated by peripheral eQTLs. If these causal associations are confirmed, m^6^A, microbial, metabolic and pyroptotic pathways—though statistically partial—could offer peripheral intervention nodes for early prevention and precision enhancement of existing therapies. It must be particularly emphasized that this study generates scientific hypotheses rather than establishing mechanisms. Prior to clinical translation, rigorous experimental validation is essential to validate the reliability of these findings.

## Author Contributions

Study concept and design: J.B., L.Z. and J.Z. Acquisition of data: L.Z., J.Z., and X.L. Analysis and interpretation of data: X.L. and X.L. Drafting of the manuscript: J.Z., X.L. and L.Z.

## Funding

This research was supported by the National Natural Science Foundation of China (Grant No. 82004285, 82505486), the Joint Science and Technology Project of the State Administration of Traditional Chinese Medicine (Grant No. GZY‐KJS‐SD‐2023‐045), and the Special Project for Clinical Research of Shandong University of Traditional Chinese Medicine (Grant No. LCKY202412). Shandong Provincial Medical and Health Science and Technology Project (Health Care Project) (Grant No. 2023BJ000011, 2023BJ000016). Shandong Provincial Traditional Chinese Medicine Science and Technology Project (Grant No. M20241811).

## Ethics Statement

The authors declare that this study utilized publicly available, de‐identified summary‐level data from previously published genome‐wide association studies. No original human participant data were collected for this study. All source datasets are publicly accessible through established repositories (e.g., FinnGen), and have been generated and shared in accordance with ethical approvals obtained by the original data collectors.

## Consent

The authors have nothing to report.

## Conflicts of Interest

The authors declare no conflicts of interest.

## Supporting information


**Data S1:** cns70840‐sup‐0001‐DataS1.xlsx.

**Data S2:** cns70840‐sup‐0002‐TableS14.docx.

## Data Availability

The data that support the findings of this study are available from the corresponding author upon reasonable request.
